# Pilot study comparing effects of infrared neuromodulation and transcranial magnetic stimulation using magnetic resonance imaging

**DOI:** 10.3389/fnhum.2025.1514087

**Published:** 2025-03-13

**Authors:** Sophia A. Bibb, Emily J. Yu, M. Fiona Molloy, John LaRocco, Patricia Resnick, Kevin Reeves, K. Luan Phan, Sanjay Krishna, Zeynep M. Saygin

**Affiliations:** ^1^Department of Psychology, The Ohio State University, Columbus, OH, United States; ^2^Department of Psychiatry and Behavioral Health, The Ohio State University Wexner Medical Center, Columbus, OH, United States; ^3^Department of Biomedical Engineering, The Ohio State University, Columbus, OH, United States; ^4^Department of Psychiatry, University of Michigan, Ann Arbor, MI, United States; ^5^Department of Psychology, Georgia State University, Atlanta, GA, United States; ^6^Department of Electrical and Computer Engineering, The Ohio State University, Columbus, OH, United States

**Keywords:** photobiomodulation, transcranial magnetic stimulation, functional magnetic resonance imaging, fractional anisotropy, resting-state connectivity

## Abstract

No prior work has directly compared the impacts of transcranial photobiomodulation (tPBM) and transcranial magnetic stimulation (TMS) on the human brain. This within-subjects pilot study compares the effects of tPBM and TMS of human somatomotor cortex on brain structural and functional connectivity. Eight healthy participants underwent four lab visits each, each visit consisting of a pre-stimulation MRI, stimulation or sham, and a post-stimulation MRI, respectively. Stimulation and sham sessions were counterbalanced across subjects. Collected measures included structural MRI data, functional MRI data from a finger-tapping task, resting state functional connectivity, and structural connectivity. Analyses indicated increased activation of the left somatomotor region during a right-hand finger-tapping task following both tPBM and TMS. Additionally, trending increases in left-lateralized functional and structural connectivity from M1 to thalamus were observed after tPBM, but not TMS. Thus, tPBM may be superior to TMS at inducing changes in connected nodes in the somatomotor cortex, although further research is warranted to explore the potential therapeutic benefits and clinical utility of tPBM.

## 1 Introduction

Pharmacological treatment of neurological disorders (e.g., Parkinson’s Disease; PD) and psychiatric disorders (e.g., Major Depressive Disorder; MDD) has had limited success and numerous undesirable side effects (Borovac, 2016; Ferguson, 2001). As such, there is a recent push to identify non-pharmacological treatments that target relevant brain regions and circuits using neuromodulation techniques such as deep brain stimulation (DBS) and transcranial magnetic stimulation (TMS). Though both techniques have had success at treating both MDD and PD, each has notable limitations that decrease its clinical utility. For example, the implementation of electrodes for DBS require risky surgery (Kantzanou et al., 2021), and the implanted pulse generators have a limited lifespan due to battery failure (Sarica et al., 2021). Meanwhile, TMS may afford greater specificity of treatment than pharmaceuticals without the invasive surgery of DBS (Hallett, 2007; Khedr et al., 2005; Lefaucheur, 2019; Schiena et al., 2021); however, TMS is limited to stimulating only the cortical surface due to rapid attenuation of the electric field as it nears the center of the brain (Deng et al., 2013; Hardwick et al., 2014). Sophia A Bibb novel method of brain stimulation, known as transcranial photobiomodulation (tPBM), may help to address these shortcomings.

Transcranial photobiomodulation involves the use of light from the visible and near infrared (NIR) spectrum at a low power density on soft tissue for therapeutic effect (Cardoso et al., 2022; Fekri et al., 2019; Zomorrodi et al., 2019). In the context of neuromodulation, tPBM is applied to the scalp and penetrates through the bone up to ∼4 cm below the cortical surface (Tedford et al., 2015), which is deeper than TMS is able to penetrate, even when using the largest TMS coils available (Deng et al., 2013). The ability to penetrate to deeper brain structures may allow clinicians to target nodes that are inaccessible using TMS, which may in turn improve clinical outcomes of treatment (Fitzsimmons et al., 2024; Reddy et al., 2023).

Prior research investigating the impact of tPBM on brain function has demonstrated that use of 600–1,070 nm wavelength tPBM improves both locomotive and cognitive behavior in both human and animal models (Fekri et al., 2019; Figueiro Longo et al., 2020; Johnstone et al., 2021; Wang et al., 2019). Clinically relevant findings in humans include improvement of cognitive function in individuals with Alzheimer’s disease (Johnstone et al., 2016) and general increase in positive affect following treatment (Barrett and Gonzalez-Lima, 2013). Although tPBM’s precise mechanisms of action are not yet clear, researchers hypothesize that the observed functional changes in brain activity are the result of either direct stimulation of distressed neurons or indirect stimulation of neurons via increased ATP production in neuronal mitochondria (Chung et al., 2012; Wang et al., 2017).

While extant research has characterized the effects of tPBM on neuronal function, no prior studies have directly compared the effects of tPBM and TMS on functional and structural connectivity between stimulated regions and connected nodes. Here, we use a multi-wavelength tPBM system to investigate the effects of 1,064 nm laser treatment on brain function and connectivity in the primary motor (M1) and primary somatosensory (S1) cortices (henceforth referenced together as the somatomotor cortex) as compared to high-frequency TMS. In this initial stage to establish proof of concept, we focused our treatment on the somatomotor cortex for two reasons: first, because motor deficits are prevalent in/comorbid to both neurological and psychiatric disorders (e.g., PD and MDD); second, because somatomotor cortex is an optimal target to compare the effects of *both* tPBM and TMS because of its proximity to the cortical surface. We asked two questions in this study. First, can tPBM alter BOLD signal in the somatomotor cortex during a finger-tapping task, and how do the effects of this stimulation compare to functional changes elicited by TMS? We investigated this question using functional task MRI. Second, how does tPBM treatment impact efferent motor pathways and connected regions? We explored this question by assessing functional and structural connectivity before and after stimulation. All exploratory analyses were conducted with the goal of identifying short-term effects of tPBM stimulation (compared to TMS and sham) within subjects.

## 2 Materials and methods

### 2.1 Participants and demographics

Ten healthy volunteers were recruited from the Ohio State University campus. Participants were required to be 18–35 years of age, native English-speakers, report normal or corrected-to-normal vision (including visual acuity, binocular depth perception, and color vision), report normal audition, no history of neurological or psychiatric diseases, and have a primary care practitioner (required by the Center for Cognitive and Behavioral Brain Imaging center). Further, participants were required to have no contraindications for MRI or TMS, no pregnancy, and not be taking psychoactive substances or medications that induce light-sensitivity. Participants were informed of any risks and provided informed consent prior to participation. Monetary compensation was provided for voluntary participation in the study. The protocol was approved by the Institutional Review Board (protocol number: IRB- 2022H0044). Two participants failed to complete all study sessions, resulting in a final sample of eight healthy participants (two female; *M* = 23.23 years, *SD* = 2.96 years).

### 2.2 Procedures for TMS and tPBM

**FIGURE 1:**
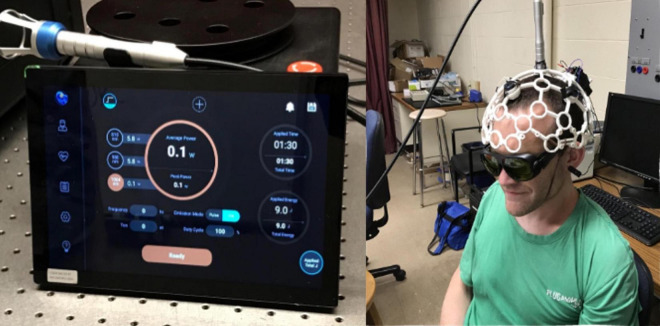
Transcranial photobiomodulation (tPBM) system and setup. **(Left)** Laser system control console. **(Right)** Participant with headset, with laser situated over the vertex of the head (Cz).

Each participant underwent four neuromodulation visits, wherein they received one of the following treatments: tPBM, sham tPBM, TMS, or sham TMS, with order counterbalanced across subject (Table 1). We included sham stimulation to control for the effects of experience and task-learning on collected brain measures. Days between adjacent lab visits varied from two to 64 days (*M* = 13 days; *SD* = 15 days). For both tPBM and sham tPBM, participants were comfortably seated facing away from the tPBM system and were instructed to keep still during stimulation. Both the participants and experimenters wore dark safety goggles during both sham and active sessions. The tPBM system (shown in Figure 1) consisted of a 1,064 nm (Zhao et al., 2022) continuous wave laser (EVO FX Tri-wavelength Class-4 Therapy Laser). The circular laser beam was positioned over the vertex of the cranium according to the 10–20 international system. Importantly, the vertex was used as the point of stimulation in order to balance the headset, as participants found it uncomfortable to wear if the laser were positioned directly over the somatomotor region; we felt comfortable doing this because previous work has demonstrated that tPBM stimulation has broad focality, making precision of application less essential for tPBM than for TMS (Wang et al., 2019). Furthermore, prior work from our group simulating the optical scattering of the tPBM laser using Monte Carlo eXtreme from MATLAB (Fang and Yan, 2019) confirmed that the bihemispheric somatomotor regions, including the right hand area of M1, fell within the range of stimulation when the laser was applied at the vertex. The laser was held in place by a modified OpenBCI 3D printed headset with a strap under the participant’s chin. Active tPBM sessions consisted of laser output of 0.6 W for 11 min for a total dosage of 396 J. Sham tPBM sessions consisted of 0.1 W for 11 min, and black electrical tape placed over the beam to assist in blocking the laser.

**TABLE 1:** Counterbalanced order of stimulation vs. sham sessions for transcranial photobiomodulation (tPBM) and transcranial magnetic stimulation (TMS) for all participants.

Participant	Session 1	Session 2	Session 3	Session 4
1	tPBM stim	tPBM sham	TMS stim	TMS sham
2	tPBM sham	TMS stim	TMS sham	tPBM stim
3	TMS sham	tPBM sham	tPBM stim	TMS stim
4	tPBM stim	TMS sham	tPBM sham	TMS stim
5	tPBM sham	tPBM stim	TMS sham	TMS stim
6	TMS stim	tPBM sham	tPBM stim	TMS sham
7	TMS sham	tPBM sham	tPBM stim	TMS stim
8	tPBM stim	TMS sham	TMS stim	tPBM sham

In all instances, stim, stimulation. Each session consisted of a pre-stimulation MRI, stimulation or sham, and a post-stimulation MRI.

Transcranial magnetic stimulation was delivered using a MagPro X100 stimulator with a Cool B6 Coil. Participants first underwent the motor cortex mapping and motor threshold determination. Single pulses of biphasic waves, starting at 30% of maximum stimulator output, were provided to a point corresponding to C3 on the participant’s head, 45° to the midsagittal line. Stimulation was titrated until 50% of trials elicited a visible contraction of the first dorsal interosseus muscles of the hand, and the pulse amplitude and location of the coil were recorded for each participant (Badran et al., 2019). The coil contained an active stimulation face, where the electromagnetic field could pass freely to the subject’s cortex, and an opposing, shielded face, with an insert that blocked the electromagnetic field during sham sessions. The faces were not marked for the subject, so the subject remained blinded to the condition. During TMS sessions, biphasic stimulation was provided to this spot using the excitatory intermittent theta burst protocol (Oberman et al., 2011) at 80% of the participant’s resting motor threshold for 600 pulses. Sham TMS consisted of the same protocol, but with the shielded face of the coil applied to the participant’s head.

### 2.3 Imaging acquisition parameters

Magnetic resonance imaging scans were conducted twice per visit, both pre- and post-stimulation or sham. Each MRI session lasted approximately 1 h, including all tasks and time getting the participant comfortably in and out of the scanner. Participants were scanned using Siemens 3T Magnetom Prisma with a 32-channel head coil. First, a high resolution (TE/TR = 2.9/2,300 ms, voxel size = 1.0 × 1.0 × 1.0 mm^3^) T1 weighted 3D magnetization-prepared rapid acquisition with gradient echo (MPRAGE) was collected. Next, 10 min of DWI data (to compute structural connectivity) were acquired using echo planar imaging (EPI; 78 slices, voxel size 2.0 × 2.0 × 20 mm^3^, 256 mm field of view, bandwidth of 1,698 Hz/Px, TR = 3,400 ms) with a multiband factor of two with b-values of 2,000 s/mm^2^, and one b = 0 volume. Diffusion weighting was isotropically distributed along 96 directions. Then, 10 min of resting state fMRI data (to define functional connectivity) was collected using echo planar imaging on all participants while awake for approximately 10 min (TE/TR = 28/2,000 ms, voxel size = 2.0 × 2.0 × 2.0 mm^3^).

### 2.4 Task design

Task fMRI scans (EPI sequence, TR = 1,000 ms, TE = 28 ms, voxel size = 2.0 × 2.0 × 3.0 mm^3^) consisted of three 3.72 min runs of a block-design finger-tapping task per MRI session (Khoury et al., 2019). Each run of the motor task was comprised of four 18 s blocks of rest and four 18 s blocks of motor action, for a total of eight blocks per run. Rest was cued by presentation of a fixation cross, wherein participants were instructed to remain still and move as little as possible. Motor action was cued by a line drawing of a hand, whereafter participants were instructed to tap either the second or third digit of their right hand on a button box (following the finger indicated on the picture) at a frequency of 1 Hz. The output from the button box was monitored to ensure that the participant remained awake and complied with the task directions. A total of six finger-tapping task runs were collected per visit—three runs pre-stimulation, and three runs post-stimulation.

### 2.5 Preprocessing and analysis of structural MRI

All structural (MPRAGE) images were preprocessed using the recon-all pipeline in FreeSurfer (FreeSurfer Wiki, 2024c) with default parameters. This pipeline includes intensity correction, skull stripping, surface co-registration, spatial smoothing, segmentation of white matter and subcortical areas, and parcellation of the cortex. All outputs were visually inspected to ensure successful reconstruction. The reconstructed images were used for multimodal registration within subject and across sessions, as well as to the group average template.

### 2.6 Preprocessing and analysis of task fMRI

First, we examined whether tPBM would increase BOLD response during a finger-tapping task (Khoury et al., 2019) and how any observed change compared to that induced by TMS. Task data preprocessing was conducted in FreeSurfer using FS-Fast and included (a) motion correction, where timepoints with greater than 1 mm total vector moment between TRs were identified and regressed out as framewise nuisance regressors, (b) distortion correction, and (c) registration to anatomical space (using FreeSurfer’s bbregister) (Greve and Fischl, 2009). For each subject, the neural activation of motor activity (β estimates and contrast maps) were computed in FreeSurfer (using FS-Fast) using a first-level general linear model (GLM) analysis. Data were detrended and smoothed (FWHM = 3 mm kernel) with a regressor entered for the contrast of interest (finger tapping) as well as six motion parameters as nuisance regressors. A block design with a standard boxcar function (events on/off) convolved with a canonical hemodynamic response function was used. The resulting contrast maps for finger tapping over baseline were used for the probabilistic maps of activation. Individual contrast maps were registered from the subject’s native space (FreeSurfer Wiki, 2024a), and then projected onto the surface of each hemisphere and then to FreeSurfer’s FSAverage space. Contrast maps were thresholded and binarized for each subject to show any voxels where the effect of stimulation was 1.3x the effect of sham stimulation, and then concatenated across subjects to create a probabilistic map of activation, with the value of voxel corresponding to the number of subjects with significant motor activation.

We further explored the effect of stimulation within each individual’s somatomotor cortex. Subject-specific functional regions of interest (fROIs) were defined in MATLAB using the Group-Constrained Subject-Specific method (Fedorenko et al., 2010). Specifically, we extracted the top 10% of activated voxels (across pre- and post-stimulation runs) within the somatomotor cortex (precentral and postcentral gyri) for the automated Desikan/Killiany segmentation (Desikan et al., 2006) in individual subject space. The percent signal change (PSC) in identified fROIs was then computed in MATLAB for the three pre-stimulation motor task runs and three post-stimulation runs using the absolute value of first-level β estimates for the motor blocks divided by the baseline (intercept) for post- and pre- runs for each session (tPBM stimulation, tPBM sham, TMS, TMS sham). The effect of stimulation on motor activation was defined as the absolute change in PSC from pre- to post-stimulation minus the change in PSC from pre- to post-sham stimulation:


((post-stimsignal-pre-stimsignal)-

(post-shamsignal-pre-shamsignal)).

One-sample *t*-tests were conducted in MATLAB to assess whether normalized PSC was significantly different from zero for both tPBM and TMS. Paired *t*-tests were then conducted to assess whether PSC due to stimulation differed within subjects between tPBM and TMS.

### 2.7 Preprocessing and analysis of structural connectivity data

Structural connectivity describes how physically connected two brain regions are by neuronal white matter tracts (Warbrick et al., 2017). Similarly to functional connectivity, alterations in white-matter have been observed in both MDD and PD (Droby et al., 2022; Liberg and Rahm, 2015); thus, we examined the absolute and relative effects of tPBM and TMS on structural connectivity of the somatomotor cortex to connected nodes. Fractional anisotropy (FA) is the measurement of the directionality of water diffusion along axons and provides an approximation of structural integrity of white matter tracts connecting two regions (Elton et al., 2023). FA image preprocessing was conducted using FDT (FMRIB’s Diffusion Toolbox), which is a part of FSL (FMRIB’s Software Library)^1^ (Behrens et al., 2003; Behrens et al., 2007). Preprocessing included an eddy current correction (eddycorrect from FDT) and brain extraction (BET from FDT). The images were then eroded and the end slices coded to zero in order to remove potential outliers. The images were then non-linearly registered to a standard template (Jenkinson et al., 2012) and thresholded at 0.2. Participant images were then concatenated into a single file for group analysis. A repeated measures ANOVA was used to calculate group statistics using threshold free cluster enhancement (TFCE) with a standard space white matter skeleton used to mask FA images.

To perform individual-subject region-to-region connectivity analyses (i.e., quantitative tractography), automated cortical parcellations were registered to each participant’s diffusion images. Image preprocessing was again conducted using FDT and included an eddy current correction (eddycorrect from FDT) and brain extraction (BET from FDT). Motor regions were used as seed regions for the analysis, while the thalamus and striatal regions were chosen as target regions. Principal diffusion directions were calculated for each voxel and probabilistic tractography was carried out using FDT with 10,000 streamline samples in each seed voxel to create a connectivity distribution to each of the target regions, while avoiding the ventricles as defined by a mask. Thus, every voxel within the motor seed region was described by a vector of connection probabilities to the target regions; we averaged these connection probabilities across voxels of the motor cortex per subject and then calculated the effect of stimulation on structural connectivity as above for functional connectivity. Inset of Figure 3 shows an example participant’s FDT_paths file, thresholded at 10% of maximum streamlines, as visualization of the tracts connecting motor to subcortex. While we had an *a priori* αlevel of 0.05, we found no differences in FA from pre- to post-tPBM stimulation at this level. Therefore, Figure 3 shows a cluster-corrected group map instead thresholded at *p* < 0.07.

### 2.8 Preprocessing and analysis of functional connectivity data

Functional connectivity describes the extent to which two brain regions coactivate and is thought to reflect both direct and indirect connections between related brain regions (Bullmore and Sporns, 2009; Logothetis, 2003). We examined how tPBM compares to TMS in modulating the brain’s functional connectivity at rest (i.e., *resting state* functional connectivity; rsFC), as disturbances in typical rsFC networks have been seen in both MDD and PD (de Kwaasteniet et al., 2013; Petersen et al., 2018; Wang et al., 2017). RsFC data preprocessing was conducted using FreeSurfer’s FS-Fast (FreeSurfer Wiki, 2024b) and included motion correction, smoothing gray matter with a 3 mm FWHM kernel, linear interpolation for spikes greater than 0.5 mm, and bandpass filtering (0.0009–0.08 Hz). The functional connectivity changes were then explored on an individual subject-level within the following regions of interest (ROIs), defined from the Desikan-Killiany automated segmentation (Desikan et al., 2006) in subject space: motor cortex, thalamus, and striatum (caudate and putamen). Correlation analyses were run for signal extracted from the motor cortex to those of the thalamus and striatum. Time courses were averaged within each region of interest, then the Pearson product correlations between the time series were computed. The *r* values were then Fisher z-transformed and the effect of stimulation on connectivity was defined using the Fisher correlations as the difference from pre- to post-stimulation over difference from pre- to post-sham stimulation.

## 3 Results

Analyses for all MRI modalities (i.e., task-based fMRI, structural connectivity, and resting state functional connectivity) were calculated based on changes in activation or connectivity after neuromodulation, normalized by changes after sham for both tPBM and TMS.

**FIGURE 2:**
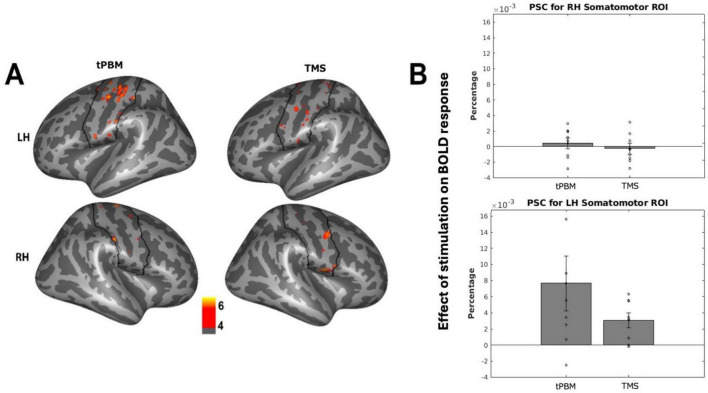
Effects of stimulation on fMRI activation. **(A)** Probabilistic map across subjects of changes in somatomotor activation after stimulation, thresholded to show effects in over 4/8 subjects. Black outline indicates somatomotor region of interest (ROI). **(B)** Individual subject changes in activation (percent signal change; PSC) of subject-specific somatomotor ROIs after stimulation, normalized by sham changes. Individual-subject data points indicated via open circles, with standard error in black lines.

We first evaluated whether tPBM and TMS induced detectable changes in BOLD response to a finger-tapping motor task in each participant’s bilateral somatomotor fROIs. tPBM stimulation of bi-hemispheric somatomotor cortex elicited changes in somatomotor activation in the left hemisphere (LH) only, controlling for changes observed in the sham sessions (see Figure 2A for probabilistic atlas of activation across participants). Stimulation of similar loci was observed in the TMS group. Extraction of subject-specific fROIs revealed that both tPBM and TMS evoked increased motor activation in the LH somatomotor fROI only [Figure 2B; tPBM: LH: *t*(7) = 2.26; *p* = 0.058; Hedge’s g = 0.710; RH: *t*(7) = 0.59; *p* = 0.57; TMS: LH: *t*(7) = 3.37; *p* = 0.012, Hedge’s g = 1.05; RH: *t*(7) = −0.36; *p* = 0.72]. There were no differences between tPBM and TMS activation in LH and RH somatomotor region fROIs [LH: *t*(7) = 1.480; *p* = 0.184; RH: *t*(7) = 1.147; *p* = 0.290].

**FIGURE 3:**
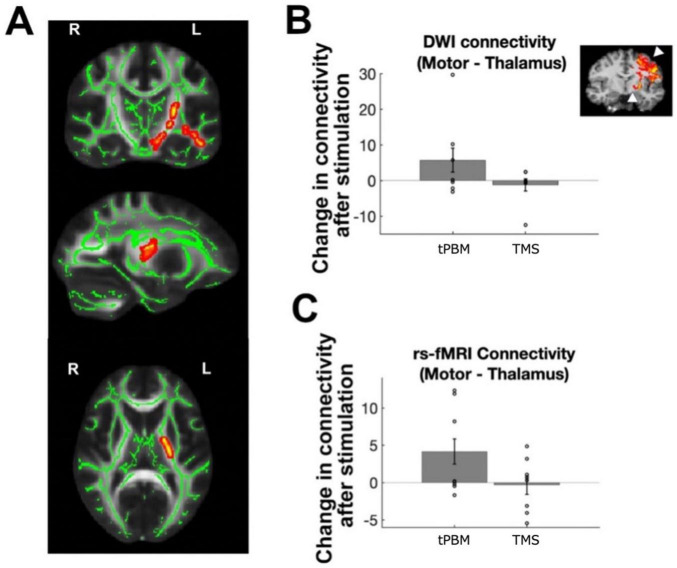
Effects of stimulation on structural and functional connectivity. **(A)** Changes in white matter FA after transcranial photobiomodulation (tPBM) stimulation, normalized by changes during sham session; cluster corrected group map at *p* < 0.07. **(B)** Changes in probabilistic tractography performed per subject to map motor to thalamic white matter connections [illustrated on representative subject in inset, with arrows indicating motor and thalamic region of interest (ROIs)]. Bar plots show changes after tPBM and transcranial magnetic stimulation (TMS) on left hemisphere (LH) motor-thalamic structural connectivity. **(C)** Changes in stimulation on LH motor-thalamic functional connectivity. Individual-subject data points indicated via open circles, with standard error in black lines.

We next evaluated changes in structural connectivity, starting with whole-brain voxelwise FA within white matter, to assess whether tPBM and TMS impacted the integrity of white matter connections between brain regions. Following tPBM stimulation, we found trending increase in FA within LH thalamic pathways only (around the anterior thalamic radiations and portions of the corticospinal tract and uncinate fasciculus; whole brain cluster correction, *p* < 0.07; Figure 3A). We found no differences between tPBM and TMS within subjects (no clusters surviving whole brain cluster correction, even at *p* < 0.1). Likewise, quantitative (probabilistic) tractography revealed a trending increase in connectivity between the LH M1 and thalamus following tPBM stimulation [LH: *t*(7) = 1.94; *p* = 0.08; RH: *t*(7) = 0.64; *p* = 0.54; Figure 3B], while TMS did not induce connectivity changes in LH [*t*(7) = 1.19; *p* = 0.28] or RH [*t*(7) = 1.79; *p* = 0.12]. There were no differences between tPBM and TMS’ effects on the LH [*t*(7) = −1.86, *p* = 0.11] or RH [*t*(7) = −0.59, *p* = 0.58] somatomotor cortices.

Functional connectivity analyses investigating the impact of stimulation on coactivation of related brain regions revealed similar results, with significant increase in LH M1-thalamus connectivity [*t*(7) = 2.35; *p* = 0.0432] but not in RH [*t*(7) = 0.78; *p* = 0.46; Figure 3C] following tPBM. TMS did not induce any significant changes in connectivity in LH [*t*(7) = 0.11; *p* = 0.92] or RH [*t*(7) = 0.48; *p* = 0.64]. There were also no differences between tPBM and TMS’ effects on functional connectivity [LH: *t*(7) = 1.84, *p* = 0.11; RH: *t*(7) = −0.93, *p* = 0.38].

## 4 Discussion

The purpose of this pilot study was to examine the functional and structural impacts of tPBM on somatomotor cortex and connected regions when compared to high-frequency TMS and sham using functional and structural MRI. Our preliminary data showed a significant increase in left-hemispheric somatomotor cortex activation during the finger-tapping task for both tPBM and TMS. We also observed trending increases in both structural and functional connectivity between left-hemispheric somatomotor regions and striatal/thalamic pathways following tPBM only.

Our findings of increased BOLD activity in LH somatomotor cortex following stimulation align with prior literature (Khedr et al., 2005; Kim and Shin, 2014; Saltmarche et al., 2017) and provide evidence that both tPBM and TMS may have a modulatory effect on BOLD activation in response to a motor task. Based on prior work, we hypothesize that areas that tPBM increased blood flow to the somatomotor cortex during the finger-tapping task by means stimulating mitochondrial respiration, resulting in increased production of the vasodilator nitrous oxide, one of the biproducts of mitochondrial respiration (Poyton and Hendrickson, 2016; Saucedo et al., 2021). While we did not collect a behavioral measure of motor task performance (e.g., speed of tapping), future work may include fMRI analyses of pre- and post-stimulation effortful motor tasks to assess change in behavioral motor function.

As aforementioned, tPBM at the vertex stimulated both left and right somatomotor cortices; however, trending change was only observed in LH somatomotor cortex. This may suggest that cortical areas active before and after stimulation, as in the finger-tapping task, may be primed for change after stimulation—though further research is needed. Additionally, our trending increases in functional and structural connectivity suggest tPBM might surpass TMS in inducing structural changes in connected nodes, a finding that is tentatively supported by extant research (Chao, 2019; Urquhart et al., 2020). However, structural changes following TMS have also been reported (Beynel et al., 2020; Schiena et al., 2021), and our findings did not reach statistical significance. Furthermore, we found no statistical difference between tPBM and TMS in subcortical connectivity. Our null findings may stem from insufficient power rather than TMS inefficacy, warranting further investigation into their differential effects on connectivity.

Indeed, one limitation of this study is the small, predominantly male sample. Although the within-subjects design enhances statistical power, there is high individual variability in tPBM and TMS effects (Dole et al., 2023; Wassermann et al., 2008); furthermore, it is unclear whether sex impacts the results of TMS and tPBM stimulation, and due to sample size, the present study is underpowered to test sex differences. Larger samples may yield stronger effect sizes and allow for investigation of sex differences in stimulation effects. The sample’s average age is also relevant, as neuroplasticity and oxidative stress change across the lifespan, potentially altering tPBM and TMS effects on brain connectivity (Chan et al., 2019; Gutiérrez-Menéndez et al., 2022; Saucedo et al., 2021). Future studies should seek to compare younger and older healthy populations. Additionally, baseline differences in connectivity and stress levels between healthy individuals and those with PDD or MDD may influence responses to tPBM (Cardoso et al., 2022; Petersen et al., 2018). Clinical samples are needed to determine whether these preliminary findings generalize to patient populations.

It is also worth noting that we observed differences between subjects in the direction and extent of change in somatomotor activation following stimulation. While we address this by normalizing every individual’s post-neuromodulation data by sham, future work investigating the impact of tPBM and TMS on neural circuitry, including the effect of stimulation on neuroinflammation, mitochondrial function, and synaptic plasticity, may help to explain the directionality and causal mechanisms behind the physiological changes that occur following tPBM and TMS in human participants (Saucedo et al., 2021; Zong et al., 2020). Furthermore, because of our small sample size, we lacked sufficient power to investigate whether there were any order effects (i.e., long-term effects of the first treatment that might have impacted subsequent treatments). Alhough we attempted to control for order effects by counterbalancing treatment order, future work specifically investigating the long-term effects of tPBM and TMS is warranted.

With regards to the tPBM stimulation, further investigation is needed to examine whether different infrared wavelengths and irradiation levels of tPBM can sufficiently target deeper brain structures, such as the ventral and dorsal striatum, which cannot be stimulated using non-invasive techniques like TMS. Finally, it is important to consider the focality of tPBM stimulation. Due to optical scattering of light as the laser passes through brain tissue, tPBM stimulates a large area of the brain with broad focality (Boas et al., 2002; Huang et al., 2021; Yuan et al., 2020). While this quality is useful for stimulating multiple related brain regions at once (i.e., somatomotor cortex) for maximum therapeutic effect, it is also important to consider the influence of off-target effects of other brain regions on observed effects on brain function.

In conclusion, the present work highlights the potential utility of tPBM to stimulate neuronal activity and increase functional and structural connectivity of the targeted area to connected nodes. In the context of clinical work, tPBM shows promise as a tool to induce neuroplasticity in diseased populations, though further work in diseased samples is necessary to corroborate our tentative findings. As the first study to directly compare tPBM and TMS using MRI, the present work serves as a useful pilot study, paving the way for future investigation of the clinical utility of tPBM for motor deficits in humans.

## Data Availability

The datasets for this article are not publicly available due to concerns regarding participant anonymity. Requests to access the datasets should be directed to the corresponding author.

## Footnotes

1
www.fmrib.ox.ac.uk/fsl
